# Supplementary material for: Children exhibit superior memory for attended but outdated information compared to adults
Source: Nat Commun. 2024 May 14;15:4058. doi: 10.1038/s41467-024-48457-0 (PMC11094159; doi:10.1038/s41467-024-48457-0)
Supplement: Supplementary file 1 — Supplementary Information [file 41467_2024_48457_MOESM1_ESM.pdf]

Supplementary Information

Supplementary Methods

In Experiment 3, we employed a task where participants had to locate a target number larger than five among distractor numbers smaller than five, with the aim of eliminating the possibility of categorization. Notably, some previous research has shown that adults could learn the consistent mapping of items to target and non-target categories even when the stimuli pertain to the same type (e.g., Letter Set 1 as targets and Letter Set 2 as distractors) after thousands of training trials[1]. However, it seems unlikely that participants could have acquired such a mapping in Experiment3, which used stimuli from the same category (i.e., numbers) as both targets and distractors and with only 11 pre-surprise trials. To provide direct evidence that participants did access the identity/magnitude of the target number in the specific task set in our study, rather than relying on categorization/mapping, we conducted Supplementary Experiments 1a and 1b using similar tasks as in Experiment 3. Twenty new adults (M_{age} = 20.95 ± 2.50 years, 16 women and 4 men) participated in Supplementary Experiment 1a and 20 new adults (M_{age} = 20.35 ± 2.25 years, 11 women and 9 men) participated in Supplementary Experiment 1b.

In Supplementary Experiment 1a, each trial started with a 500-ms fixation display. After a 500-ms blank interval, a black Arabic number (between 1 and 9 except for 5, 0.6°×0.8°) was presented at the center of the screen, and participants were asked to judge whether this number was larger or smaller than five by pressing one of two keys as quickly and accurately as possible. The trial ended with a response or after 2000 ms elapsed, and

the next trial started after an interval of 1500 ms. Participants completed two blocks of 96 trials, one with the "larger" response assigned to the "J" key and the "smaller" response assigned to the "F" key, and one with the reversed assignment. The order of the blocks was counterbalanced among participants. The eight numbers were presented with equal frequency and in a randomized order. There were 16 practice trials before each block. This experiment was pre-registered on Open Science Framework [https://osf.io/qk5x8] (March 27, 2023) and performed with no deviations from the pre-registration.

For Supplementary Experiments 1a and 1b, the accuracy was near ceiling (>98%) and mean reaction time (RT) was used as the dependent measure. Only trials with correct responses were included in RT analysis. We used the classical distance effect to evaluate the quantitative representation of numbers, which refers to the observed phenomenon that it is more difficult to compare two numbers if they are close than if they are far apart[2]. Accordingly, if the quantitative information is represented in this task, the RT will decrease as the numerical distance (1-4 units) from the reference number (i.e., 5) increases. For instance, the reaction to the number 9 would be faster than the reaction to the number 6, because 9 is further away from the reference number 5, making it easier for the participants to judge. Otherwise, if participants complete this task through categorization without identifying the magnitude of number, no such distance effect should be observed. As shown in Supplementary Figure 1a, we observed a clear distance effect. A one-way repeated-measures analysis of variance (ANOVA) yielded a significant main effect of the numerical distance, $F(2.144, 40.745)=34.822$, $p<0.001$, $\eta_p^2=0.647$. Moreover, there was a significant linear trend where RT decreased as numerical distance increased,

$F(1,19)=82.879$, $p<0.001$, $\eta_p^2=0.814$. This linear trend was consistent for both numbers smaller than five ($F(1,19)=19.677$, $p<0.001$, $\eta_p^2=0.509$) and numbers larger than five ($F(1,19)=22.617$, $p<0.001$, $\eta_p^2=0.543$).

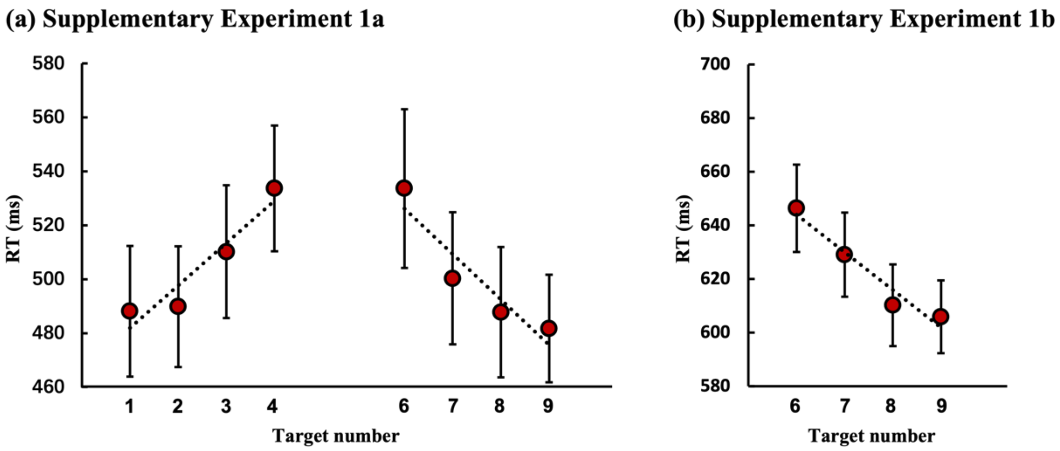

Supplementary Figure 1. RT results of Supplementary Experiments 1a and 1b. Error bars represent standard error of the mean. Source data are provided as a Source Data file.

To further confirm that numerical quantity was accessed in the current study's particular task set, we measured the distance effect in Supplementary Experiment 1b, where participants were asked to locate a target number larger than five among three distractor numbers smaller than five (a similar task as in Experiment 3). The procedure of Supplementary Experiment 1b was identical to Experiment 3 except as follows. Participants were asked to report the location of the target number immediately after the stimuli were shown, by pressing a corresponding key ("F", "V", "J", or "N") as quickly and accurately as possible. The stimuli display ended with a response or after 2000 ms elapsed, and the next trial started after an interval of 1500 ms. Participants completed eight practice trials before completing two formal blocks of 80 trials. The four target numbers were presented with equal frequency in each location and in a randomized order. This

experiment was pre-registered on Open Science Framework [https://osf.io/5edz6] (March 30, 2023) and performed with no deviations from the pre-registration.

As shown in Supplementary Figure 1b, we again found a clear distance effect in this task set. There was a significant main effect of the numerical distance, $F(2.115,40.193)=8.950$, $p<0.001$, $\eta_p^2=0.320$, and a significant linear trend where RT decreased as numerical distance increased, $F(1,19)=32.530$, $p<0.001$, $\eta_p^2=0.631$. These results yielded evidence that the quantitative representation of the target number was activated in this task set.

References

1. R. M. Shiffrin, W. Schneider, Controlled and automatic human information processing: II. Perceptual learning, automatic attending and a general theory. *Psychol. Rev.* **84**, 127–190 (1977).
2. R. S. Moyer, T. K. Landauer, Time required for judgements of numerical inequality. *Nature* **215**, 1519–1520 (1967).
