# Supplementary material for: Children exhibit superior memory for attended but outdated information compared to adults
Source: Nat Commun. 2024 May 14;15:4058. doi: 10.1038/s41467-024-48457-0 (PMC11094159; doi:10.1038/s41467-024-48457-0)
Supplement: Supplementary file 3 — Reporting Summary [file 41467_2024_48457_MOESM3_ESM.pdf]

nature portfolio

Corresponding author(s): Hui Chen

Last updated by author(s): Jan 28, 2024

Reporting Summary

Nature Portfolio wishes to improve the reproducibility of the work that we publish. This form provides structure for consistency and transparency in reporting. For further information on Nature Portfolio policies, see our Editorial Policies and the Editorial Policy Checklist.

Statistics

For all statistical analyses, confirm that the following items are present in the figure legend, table legend, main text, or Methods section.

n/a	Confirmed	
☐	☒	The exact sample size (*n*) for each experimental group/condition, given as a discrete number and unit of measurement
☐	☒	A statement on whether measurements were taken from distinct samples or whether the same sample was measured repeatedly
☐	☒	The statistical test(s) used AND whether they are one- or two-sided<br>*Only common tests should be described solely by name; describe more complex techniques in the Methods section.*
☒	☐	A description of all covariates tested
☐	☒	A description of any assumptions or corrections, such as tests of normality and adjustment for multiple comparisons
☐	☒	A full description of the statistical parameters including central tendency (e.g. means) or other basic estimates (e.g. regression coefficient) AND variation (e.g. standard deviation) or associated estimates of uncertainty (e.g. confidence intervals)
☐	☒	For null hypothesis testing, the test statistic (e.g. F, t, r) with confidence intervals, effect sizes, degrees of freedom and P value noted<br>*Give P values as exact values whenever suitable.*
☐	☒	For Bayesian analysis, information on the choice of priors and Markov chain Monte Carlo settings
☒	☐	For hierarchical and complex designs, identification of the appropriate level for tests and full reporting of outcomes
☐	☒	Estimates of effect sizes (e.g. Cohen's *d*, Pearson's *r*), indicating how they were calculated

Our web collection on statistics for biologists contains articles on many of the points above.

Software and code

Policy information about availability of computer code

Data collection	All experiments were programmed and executed using MATLAB 2014a with the Psychtoolbox-3 functions.
Data analysis	The data analysis in these experiments was conducted in JASP 0.17.3. The analysis code for original eye-movement data was custom developed in MATLAB 2014a.

For manuscripts utilizing custom algorithms or software that are central to the research but not yet described in published literature, software must be made available to editors and reviewers. We strongly encourage code deposition in a community repository (e.g. GitHub). See the Nature Portfolio guidelines for submitting code & software for further information.

Data

Policy information about availability of data

All manuscripts must include a data availability statement. This statement should provide the following information, where applicable:

- Accession codes, unique identifiers, or web links for publicly available datasets
- A description of any restrictions on data availability
- For clinical datasets or third party data, please ensure that the statement adheres to our policy

The datasets generated and analyzed during the current study are available on the Open Science Framework (OSF) [https://osf.io/qhy3r/].

Research involving human participants, their data, or biological material

Policy information about studies with human participants or human data. See also policy information about sex, gender (identity/presentation), and sexual orientation and race, ethnicity and racism.

Reporting on sex and gender	We collected the sex information for each participant through self-reporting of their sex (binary choice: male or female) prior to the commencement of the experiment. Sex was not considered as a factor in this study design because we tested the development of a general cognitive ability and no prior studies have reported the sex effect on attribute amnesia.
Reporting on race, ethnicity, or other socially relevant groupings	All participants were Chinese adult students or children. The current study complies with ethical regulations for research on human participants.
Population characteristics	See above.
Recruitment	The adults were recruited from Zhejiang University in exchange for course credits or a monetary payment, and all of them reported normal or corrected-to-normal visual acuity. The children were recruited from a kindergarten in Hangzhou, China, with no reported vision, hearing, or developmental issues. The sampling procedure was convenience sampling. For adults, a posting was put up on the subject pool and students volunteered to participate; for children, the participants were randomly chosen with agreements from parents and teachers.
Ethics oversight	Informed consent was obtained from the parents of the children and the adult participants themselves prior to each experiment in accordance with the institutional review board of Zhejiang University.

Note that full information on the approval of the study protocol must also be provided in the manuscript.

Field-specific reporting

Please select the one below that is the best fit for your research. If you are not sure, read the appropriate sections before making your selection.

☐ Life sciences ☒ Behavioural & social sciences ☐ Ecological, evolutionary & environmental sciences

For a reference copy of the document with all sections, see nature.com/documents/nr-reporting-summary-flat.pdf

Behavioural & social sciences study design

All studies must disclose on these points even when the disclosure is negative.

Study description	This was a quantitative experimental study. Two groups of participants(adults/children) were asked to complete the attribute amnesia task. For example, participants were asked to find a target letter among three distractor digits and report its location. In one critical trial (i.e., surprise trial), they were unexpectedly asked to report the identity of the target letter (i.e., the key feature). The performance (accuracy) in reporting the key feature in the surprise test between two groups would be compared.
Research sample	The adults were recruited from Zhejiang University in exchange for course credits or a monetary payment, and all of them reported normal or corrected-to-normal visual acuity. The children were recruited from a kindergarten in Hangzhou, China, with no reported vision, hearing, or developmental issues.The mean age and sex characteristics of the participants in each experiment were as follows: Experiment 1, adults: Mage = 18.95 ± 1.00 years (13 women and 7 men), children: Mage = 5.75 ± 0.85 years (8 girls and 12 boys); Experiment 2, adults: Mage = 20.50 ± 3.50 years (18 women and 2 men), children: Mage = 6.10 ± 0.55 years (9 girls and 11 boys); Experiment 3, adults: Mage = 24.20 ± 2.78 years (5 women and 15 men), children: Mage = 5.95 ± 0.51 years (5 girls and 15 boys); Experiment 4, adults: Mage = 19.80 ± 1.79 years (9 women and 11 men), children: Mage = 6.00 ± 0.56 years (9 girls and 11 boys); Experiment 5, adults: Mage = 21.76 ± 3.02 years (28 women and 22 men), children: Mage = 5.48 ± 0.61 years (21 girls and 29 boys); Supplementary Experiment 1a, Mage = 20.95 ± 2.50 years (16 women and 4 men); Supplementary Experiment 1b, Mage = 20.35 ± 2.25 years (11 women and 9 men). There was no overlap in participants across the experiments.<br>Our sample is considered representative of the target population because 1)Participants were chosen through a random and unbiased selection process; 2)The participants were recruited over an extensive timeframe spanning from 2017 to 2023; 3) The age of the children in all experiments was similar, centered around 5-6 years. However, it's important to note that all children in the study were from Hangzhou, China, and we recognize that the generalizability of our findings may be influenced by cultural or educational differences in other regions or countries.<br>We opted for a convenience sampling approach when selecting our study sample. Convenience sampling was chosen for its practicality and efficiency, allowing us to readily access participants who met the criteria for our study. This decision was guided by the recognition that the attribute amnesia effect has been widely observed and replicated in previous research and thus we deemed the investigation of memory selection to be a fundamental aspect of cognitive function that is likely to manifest consistently.
Sampling strategy	The sampling procedure was convenience sampling. The sample sizes were predetermined by power analysis using G*Power 3.1 (Faul, Erdfelder, Lang, & Buchner, 2007).
Data collection	All data was collected and stored on experimental computers running MATLAB software with the Psychophysics Toolbox extension, on a 14-inch laptop monitor. The adults were tested in a quiet room in Zhejiang University and the children were tested in a quiet room in the kindergarten. The participants signed the informed consent forms as they arrived. Then they were assigned a participant ID and sat in front of the computer at a viewing distance of approximately 50 cm. In Experiment 4, an EyeLink II system (EyeLink

	Portable Duo, SR Research, Mississauga, Ontario, Canada) recorded eye position monocularly from the left eye with a sample rate of 1000 Hz. At the beginning of the experiment, the eye tracker was calibrated using a 5-point calibration procedure.<br>No one was present besides the participant and the researcher. The participants, but not the researcher, were blinded to the experimental condition and/or the study hypothesis. The data were collected by various researchers, including the authors and others mentioned in the acknowledgments.
Timing	All of the data was collected between 5/4/2017 to 11/9/2023.
Data exclusions	For eye movement data analysis, trials with no eye movement or false responses in the location task were excluded. The exclusion of false responses in the location task removed 2% trials in adults and 4% trials in children. The exclusion of trials with no eye movements removed 18% trials in adults and 2% trials in children. The relatively higher proportion of excluded trials with no eye movements in adults was caused by several individual subjects (5 adults made no eye movement in >30% trials, while the exclusion proportion was only 6% for the left adults). As we have no strong reason to exclude these subjects, and more importantly, both the qualitative pattern and the statistical significance of results remained the same regardless of whether these subjects were excluded, we reported the results including them in the manuscript.<br>For Supplementary Experiments 1a and 1b, only trials with correct responses were included (>98%) in RT analysis.
Non-participation	One adult and two children in Experiment 4 were excluded due to failed eye-tracking data recoding. One adult in Experiment 4 and one adult in Experiment 5 were excluded for extremely poor performance (<60%) in reporting the location in pre-surprise trials. All removed participants were replaced by new valid participants to achieve the planed sample size in each experiment.
Randomization	Each experiment included one group of adults and one group of children. Participants were not allocated to other experimental groups.

Reporting for specific materials, systems and methods

We require information from authors about some types of materials, experimental systems and methods used in many studies. Here, indicate whether each material, system or method listed is relevant to your study. If you are not sure if a list item applies to your research, read the appropriate section before selecting a response.

Materials & experimental systems

n/a	Involved in the study
☒	☐ Antibodies
☒	☐ Eukaryotic cell lines
☒	☐ Palaeontology and archaeology
☒	☐ Animals and other organisms
☒	☐ Clinical data
☒	☐ Dual use research of concern
☒	☐ Plants

Methods

n/a	Involved in the study
☒	☐ ChIP-seq
☒	☐ Flow cytometry
☒	☐ MRI-based neuroimaging
